# A case–control evaluation of 143 single nucleotide polymorphisms for breast cancer risk stratification with classical factors and mammographic density

**DOI:** 10.1002/ijc.32541

**Published:** 2019-07-13

**Authors:** Adam R. Brentnall, Elke M. van Veen, Elaine F. Harkness, Sajjad Rafiq, Helen Byers, Susan M. Astley, Sarah Sampson, Anthony Howell, William G. Newman, Jack Cuzick, Dafydd Gareth R. Evans

**Affiliations:** ^1^ Centre for Cancer Prevention, Wolfson Institute of Preventive Medicine, Charterhouse Square, Barts and The London Queen Mary University of London London United Kingdom; ^2^ Division of Evolution and Genomic Sciences, School of Biological Sciences, Faculty of Biology, Medicine and Health University of Manchester, Manchester Academic Health Science Centre Manchester United Kingdom; ^3^ Prevention Breast Cancer Centre and Nightingale Breast Screening Centre University Hospital of South Manchester Manchester United Kingdom; ^4^ Division of Informatics, Imaging and Data Sciences, Faculty of Biology, Medicine and Health University of Manchester Manchester United Kingdom; ^5^ Manchester Academic Health Science Centre University of Manchester Manchester United Kingdom; ^6^ School of Public Health, Epidemiology & Biostatistics Imperial College London London United Kingdom; ^7^ The Christie NHS Foundation Trust Manchester United Kingdom; ^8^ Manchester Centre for Genomic Medicine Manchester University NHS Foundation Trust Manchester United Kingdom; ^9^ Manchester Breast Centre, Manchester Cancer Research Centre University of Manchester Manchester United Kingdom

**Keywords:** risk prediction, risk stratification, breast cancer, SNPs, Tyrer–Cuzick, breast density

## Abstract

Panels of single nucleotide polymorphisms (SNPs) stratify risk for breast cancer in women from the general population, but studies are needed assess their use in a fully comprehensive model including classical risk factors, mammographic density and more than 100 SNPs associated with breast cancer. A case–control study was designed (1,668 controls, 405 cases) in women aged 47–73 years attending routine screening in Manchester UK, and enrolled in a wider study to assess methods for risk assessment. Risk from classical questionnaire risk factors was assessed using the Tyrer–Cuzick model; mean percentage visual mammographic density was scored by two independent readers. DNA extracted from saliva was genotyped at selected SNPs using the OncoArray. A predefined polygenic risk score based on 143 SNPs was calculated (SNP143). The odds ratio (OR, and 95% confidence interval, CI) per interquartile range (IQ‐OR) of SNP143 was estimated unadjusted and adjusted for Tyrer–Cuzick and breast density. Secondary analysis assessed risk by oestrogen receptor (ER) status. The primary polygenic risk score was well calibrated (O/E OR 1.10, 95% CI 0.86–1.34) and accuracy was retained after adjustment for Tyrer–Cuzick risk and mammographic density (IQ‐OR unadjusted 2.12, 95% CI% 1.75–2.42; adjusted 2.06, 95% CI 1.75–2.42). SNP143 was a risk factor for ER+ and ER− breast cancer (adjusted IQ‐OR, ER+ 2.11, 95% CI 1.78–2.51; ER− 1.81, 95% CI 1.16–2.84). In conclusion, polygenic risk scores based on a large number of SNPs improve risk stratification in combination with classical risk factors and mammographic density, and SNP143 was similarly predictive for ER‐positive and ER‐negative disease.

## Abstract 2

**What's new?**

Panels of single nucleotide polymorphisms (SNPs) stratify risk for breast cancer in women from the general population, but studies are needed to assess their use in a fully‐comprehensive model including classical questionnaire risk factors, mammographic density, and more than 100 SNPs associated with breast cancer. In this study, the predictive ability of a predefined panel of 143 SNPs was assessed after adjustment for questionnaire risk factors and mammography density using women from a U.K. cohort. The panel showed substantial improvement in risk stratification in combination with classical risk factors and mammographic density, for both oestrogen receptor‐positive and negative breast cancer.

**Abbreviations**
aAUC: adjusted area under the curve (adjusted concordance index)
DCIS: ductal carcinoma *in situ*
ER: oestrogen receptor
IQ‐OR: interquartile range odds ratio
IQR: interquartile range
OR: odds ratio
PRS: polygenic risk score
SNP: single nucleotide polymorphism
VAS: visual analogue scale

## Introduction

Over the past few decades, there has been increasing interest in individual risk assessment for breast cancer.^1^, ^2^, ^3^, ^4^, ^5^ Motivations for this include the identification of individuals at extremely high risk who would be potential candidates for risk‐reducing surgery or preventive therapy;^6^ delineation of populations at moderately enhanced risk who might benefit from enhanced screening;^7^ and more recently, identification of populations at sufficiently low risk as to require minimal, if any, screening.^8^ Breast cancer has a well‐established link with hormone exposure, in addition to a growing body of knowledge on genetic risk factors.^9^, ^10^, ^11^ While existing risk models have shown a degree of accuracy in prediction it is clear that there is substantial room for improvement, particularly for hormone‐receptor‐negative disease.^12^, ^13^, ^14^

It has been shown that panels of the earliest single nucleotide polymorphism (SNP) markers to be identified aid breast cancer risk stratification in Caucasian women, which is maintained after accounting for classic risk factors and mammographic density.^15^, ^16^ Progress on their use in risk assessment has largely arisen from a collaborative project (Breast Cancer Association Consortium [BCAC]), whose recent results have now confirmed the genome‐wide significance of more than 150 SNPs for all breast cancer, and identified several of these as being associated with oestrogen receptor (ER)‐negative disease.^17^

Relatively little work has been done to assess the ability of SNP panels to stratify risk in combination with classical risk factors and mammographic density, and has involved panels of up to 77 SNPs.^15^, ^16^ The aim of this case–control study was to assess the ability of a more comprehensive panel of SNPs in order to stratify risk beyond that achieved from classical risk factors and mammographic density in a sample of women attending routine screening in the UK. Our focus was on a panel that has each reached individual genome‐wide significance for association with risk of breast cancer.^17^ A secondary aim was to assess the ability of the polygenic risk score (PRS) to predict risk by ER status.

## Materials and Methods

### Patients

A study was run within the Greater Manchester National Health Service Breast Screening Program to identify suitable models to assess breast cancer risk in population settings (predicting risk of cancer at screening [PROCAS]).^18^ Women aged 47–73 years were invited once and recruited between October 2009 and June 2015 at the time of attendance for mammographic screening. Breast cancer risk factors were self‐reported by the women *via* completion of a two‐page paper questionnaire. Women were excluded from PROCAS if they had been diagnosed with breast cancer before completing the questionnaire; cancers detected as a result of the screening test were included. In total, 57,902 women were enrolled in the PROCAS study.^18^ Unaffected women in the cohort who lived within the smaller defined Withington area (South Manchester) were subsequently invited to participate in an additional risk assessment study using DNA extracted from a saliva sample; all women with breast cancer diagnosed after completion of the questionnaire were invited to provide saliva samples and participate as cases and saliva samples were obtained from 9,956 women.^16^

Breast cancer diagnosis (invasive or ductal carcinoma *in situ* [DCIS]) was at the entry screen (10/2009–06/2015) or subsequently before January 5, 2017, and was ascertained through monthly updates from (National Health Service) Breast Screening Systems. DCIS was included because it is a precursor to invasive breast cancer that is detected through screening programs, and it is clinically relevant because women are offered treatment.

Saliva samples were collected between 10/2009 and 12/2013, close to but after the time of the woman's screening visit (median 1.1 years after, interquartile range [IQR] 0.2–1.1 years).

Mammographic density at entry to the cohort was estimated independently by two readers using a visual analogue scale (VAS) for breast density, where each mammographic view was scored on a linear scale ranging from 0% to 100%. The primary measure of mammographic density was the mean percentage from two readers and four mammogram views. Only the contralateral breast was used in analysis for women with breast cancer. The derived mean percent density was adjusted for BMI and age and reported as a “density residual”, being the observed minus expected density based on BMI and age.^19^ Women with bilateral cancer on the prevalent study screen, or with breast implants, or with no assessable VAS score were excluded from the analysis. The Tyrer–Cuzick 10‐year risk (v6) was based on classical risk factors from the questionnaire self‐reported at entry.^3^

The study was approved by the North Manchester Research Ethics Committee (ref. 09/H1008/81) and written informed consent was obtained from each participant.

### Study design

In total, 2,191 women were selected for this case–control study from 9,956 women who provided saliva samples. These included all cases without a previous diagnosis of breast cancer at entry to PROCAS and a sample of unaffected controls who were matched on age (±12 months) and date (within 1 month) and type of first mammogram (analogue/digital). Approximately four unaffected individuals were included per case. Of the 2,191 women available for this case–control study, 58 were excluded (49 controls, 9 cases) because they failed quality control based on the full assay (see “Assay methods” section), and a further 41 (25 controls and 16 cases) because their SNP call rate for the 143 SNPs in the PRS (see section “Statistical analysis methods”) was <95%. In addition, 19 were excluded (8 controls, 11 cases) because mammographic density measurements were unavailable. Therefore, complete data from 1,668 controls and 405 cases were available for analysis.

### Assay methods

Saliva samples were collected using Oragene saliva lysate tubes (DNA Genotek Inc., Ottawa, Ontario, Canada) and DNA extraction was performed using Gen‐Probe extraction. SNP genotyping was undertaken using a custom Illumina genotyping platform which was specifically designed for the Collaborative Oncological Gene–Environment Study (COGS) consortium (OncoArray: http://epi.grants.cancer.gov/oncoarray). Samples were assayed in two batches and included 31 internal controls. Genotyping and quality control were performed as previously described^17^ but without exclusions due to ancestry.

### Statistical analysis methods

The primary PRS was derived beginning with the 172 SNPs listed in Supporting Information Tables^17^ previously linked with breast cancer risk. Twenty‐three SNPs were not included in the analysis because they were not present on the OncoArray and there was no SNP in tight linkage disquilibrium (*R* ^2^ > 0.9 from the LDLink tool^20^) leaving 149 SNPs. Two of these SNPs failed for all samples and were replaced by proxies (rs62355902 replaced by kgp3323585; rs6122906 replaced by rs746427). Five SNPs were excluded because their call rate in the sample was less than 98%, leaving 144 SNPs (Supporting Information Table S7). One further SNP was excluded due to high correlation with another included SNP (rs2981578 removed correlated with rs2912779 at 0.77; the SNP with the smaller *p*‐value from the prior meta‐analysis was selected). Assay quality was supported by testing Hardy–Weinberg equilibrium for each SNP in cases and controls, by comparing the observed number of homozygotes against expected using a binomial distribution (Supporting Information Table S8).

Per allele risks were taken for each SNP based on a combined meta‐analysis estimate (the GWAS, iCOGS and OncoArray study estimate from Ref. 17), leading to overall breast cancer risk based on 143 SNPs. The PRS was formed by multiplying the per‐allele odds ratio (OR) for each SNP, normalised by the average risk expected in the populations based on the assumed allele frequency, as earlier.^16^, ^21^, ^22^ Two ER‐specific risk scores were formed using a subset of the 143 SNPs that achieved genome‐wide significance (*p* < 10^−8^) for the ER‐specific subtype. There were 81 SNPs at genome‐wide significance for ER+ breast cancer risk, and 20 SNPs for ER− disease. For comparison with an earlier PRS, we used SNPs at the 18 loci previously reported to form another risk score (SNP18).^16^, ^22^ Sensitivity analysis included consideration of a less stringent criteria for the ER‐specific risks (*p* < 10^−5^), with 118 ER+ SNPs and 36 ER− SNPs.

Predictive ability was assessed using 95% Wald confidence intervals of the OR for a unit change in the IQR of the PRS in controls, adjusted for (1) age, or (2) age, the natural logarithm of 10‐year risk from the Tyrer–Cuzick model and mammographic density. Calibration of the observed (O) to the expected (E) PRS OR was estimated using the log score regression coefficient so that O/E = 1 would indicate perfect calibration, and further inspected by decile, with confidence intervals following Wilson's method for the binomial parameter. The change in likelihood ratio χ^2^ statistics when adding the PRS was used to measure statistical information. Adjusted concordance indices (aAUC) were obtained by regressing the PRS on the adjustment factors in controls, and using the residuals from this model to compute an area under the receiver operating curve, with empirical bootstrap confidence intervals.^23^ Subgroup analysis assessed the predictive ability of SNP scores using groups of 20 SNPs, ordered by their *p*‐value from Ref. 17. A *post hoc* subgroup analysis also assessed the difference between invasive breast cancer and DCIS.

Results were considered in the context of the ability of models to stratify breast cancer risk assuming independence between the Tyrer–Cuzick model, mammographic density and PRSs as justified by an earlier analysis in a wider cohort.^16^ The main focus was on a high‐risk group (>8% 10‐year projected risk, clinically relevant in the UK^24^), and a low‐risk group (<1.4% 10‐year risk; slightly less than the average risk for a woman aged 40 years).

All analysis was undertaken in the statistical software R version 3.4.1.^25^

### Data availability statement

The data that support the findings of our study are available from the corresponding author upon reasonable request.

## Results

The majority of women were older than 59 years and were overweight (Table 1). Age was well matched in cases and controls following the study design (Table 1). There were also only small differences between cases and controls for several of the classical risk factors, including family history (Table 1). This was partly because controls who donated saliva were more likely to have a family history than the wider cohort (Supporting Information Tables S1 and S2). A low level of missing data in the questionnaire fields was broadly consistent with the wider cohort (Supporting Information Tables S1 and S2).

**Table 1:** Summary of breast cancer risk factor statistics for cases and controls

Risk factor	Control	Case	*p*
(a) Continuous risk factors (median, IQR)			
Age (years)	60 (54–65)	60 (53–65)	0.48
Age first child (parous, years)	24 (19–27)	24 (19–28)	0.8
BMI (kg/m^2^)	25.9 (23.1–29.8)	26.5 (23.9–30.3)	0.005
Density (%)	26.0 (14.5–38.7)	29.5 (18.8–42.0)	<0.001
Density residual	0.01 (−0.65–0.66)	0.35 (−0.33–1.02)	<0.001
Tyrer–Cuzick model 10 years risk (%)	2.87 (2.26–3.70)	3.03 (2.35–4.22)	0.006
(b) Binary (*n*, %)			
First‐degree relative (yes, %)	225 (13.5%)	65 (16.0%)	0.21
Parous (yes, %)	1,401 (84.0%)	338 (83.5%)	0.8
White (yes, %)	1,541 (96.3%)	375 (95.9%)	0.8

*p* univariate comparison between cases and controls: continuous risk factor by Wilcoxon test; binary by chi‐square test (with continuity correction); missing data excluded (see Supporting Information).

There was very little association between the PRSs and other risk factors. The primary PRS had a very weak correlation with 10 years risk from the Tyrer–Cuzick model (Spearman 0.034, *p* = 0.169) and the mammographic density residual (0.047, *p* = 0.058).

**Figure 1:**
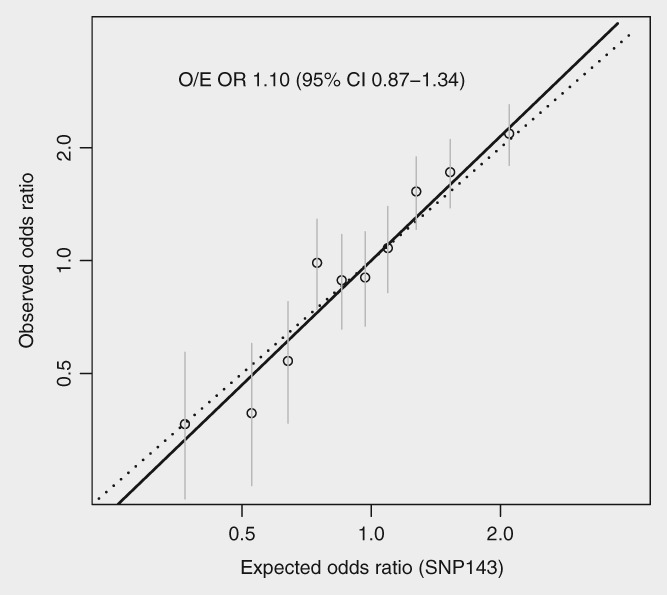
Calibration of the primary polygenic risk score (unadjusted). Points are observed and expected odds ratios by decile, the fit from a logistic regression (—) is also shown (see Supporting Information Table S3). O/E OR: a calibration coefficient for the observed (O) divided by expected (E) odds ratio (OR), or fitted slope of the line (—).

Calibration of the primary PRS appeared adequate (O/E OR 1.10 (95% CI 0.86–1.34), median log score controls −0.14 (IQR −0.50 to 0.18), Table 2). Calibration is visualised by inspecting the observed to expected ORs (O/E OR) shown in Figure 1. Predictive ability decreased by only a small amount after adjustment for the classical risk factors in the Tyrer–Cuzick model and mammographic density (interquartile range OR [IQ‐OR] 2.12, 95% CI 1.81–2.49 *vs*. adjusted 2.06, 95% CI 1.75–2.42). The primary PRS predicted similarly well for ER+ and ER− breast cancer (adjusted IQ‐OR, ER+ 2.11 (95% CI 1.78–2.51), ER− 1.81 (1.16–2.84)). The ER‐subtype PRSs performed similarly to the main polygenic score (adjusted IQ‐OR SNP‐ER+, ER+ cancer, 1.96 [95% CI 1.67–2.30]; SNP‐ER−, ER− cancer, 2.23 [1.53–3.26]), although there was some evidence that the ER− PRS underestimated relative risks (adjusted calibration coefficient 1.94, 95% CI 1.02–2.86), that is, it was more predictive than expected.

**Table 2:** Predictive information in three SNP scores (SNP143 breast cancer, SNP‐ER+ for ER+ breast cancer, SNP‐ER− for ER− breast cancer) and by endpoint (all breast cancer, ER+, ER−)

Risk score and endpoint	Controls^1^ (Median, IQR)	Cases^1^ (Median, IQR)	Adjustment^2^	IQ‐OR (95% CI)	LR‐Δχ^2^(df = 1)	Calibration (95% CI)	aAUC (95% CI)
SNP143 Breast cancer (405 cases)	−0.14 (−0.50–0.18)	0.10 (−0.21–0.44)	(i)	2.12 (1.81–2.49)	90.2	1.10 (0.86–1.34)	0.65 (0.62–0.68)
			(ii)	2.06 (1.75–2.42)	80.8	1.06 (0.82–1.29)	0.64 (0.61–0.67)
SNP143 ER+ (353 cases)	−0.14 (−0.50–0.18)	0.11 (−0.19–0.45)	(i)	2.17 (1.83–2.58)	85.8	1.13 (0.88–1.38)	0.66 (0.63–0.69)
			(ii)	2.11 (1.78–2.51)	77.5	1.09 (0.84–1.34)	0.65 (0.62–0.68)
SNP143 ER− (39 cases)	−0.14 (−0.50–0.18)	0.17 (−0.35–0.35)	(i)	1.86 (1.18–2.91)	7.4	0.90 (0.25–1.56)	0.63 (0.54–0.72)
			(ii)	1.81 (1.16–2.84)	6.9	0.87 (0.21–1.53)	0.63 (0.54–0.71)
SNP‐ER+ ER+ (353 cases)	−0.17 (−0.50–0.17)	0.13 (−0.25–0.42)	(i)	2.01 (1.71–2.36)	77.6	1.03 (0.80–1.27)	0.65 (0.61–0.68)
			(ii)	1.96 (1.67–2.30)	70.9	1.00 (0.76–1.24)	0.64 (0.61–0.67)
SNP‐ER−/ER− (39 cases)	−0.06 (−0.26–0.15)	0.12 (0.00–0.35)	(i)	2.22 (1.52–3.24)	16.5	1.93 (1.02–2.84)	0.69 (0.61–0.77)
			(ii)	2.23 (1.53–3.26)	16.6	1.94 (1.02–2.86)	0.69 (0.61–0.77)

1 Natural logarithm SNP score (odds ratio).

2 Adjusted for (*i*) age or (*ii*) fully adjusted for age, the natural logarithm 10 years Tyrer–Cuzick model risk and mammographic density.

Abbreviations: IQ‐OR, odds ratio per interquartile range in controls; LR‐Δχ^2^ change in likelihood‐ratio χ^2^ statistic when adding the SNP score to the logistic regression; aAUC area under the adjusted receiver operating characteristic.

**Figure 2:**
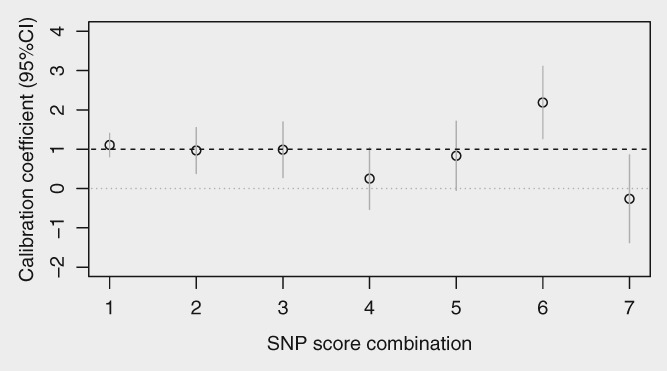
Calibration (95% CI) of the primary polygenic risk score (unadjusted) split into subscores of 20 SNPs ordered by the overview *p*‐value for each SNP (1 = top 20 [SNP1–20] predictive SNPs, 2 = next 20 [SNP21–40], similarly 3–6 and 7 = least predictive SNPs [SNP121–143]).

Most predictive information was contained in the 20 SNPs with the smallest meta‐analysis *p* values (Supporting Information Tables S5 and S8). The *a priori* top 20 SNPs contributed a LR‐χ^2^ of 55.4 compared to 90.2 for all 143 SNPs (i.e. 61% of the information). There was little trend apparent in calibration by order of the *a priori* significance in Figure 2. The top 20 SNPs contributed a similar degree of information to an earlier risk score based on 18 SNPs (adjusted IQ‐OR, top 20: 1.71 [95% CI 1.47–1.99], SNP18: 1.68 [1.44–1.96]; Supporting Information Tables S5 and S8);^16^ 13/18 loci from SNP18 were included in the top 20.

Incorporating the PRS with the Tyrer–Cuzick model and mammographic density had a substantial impact on the number of women categorised into high‐ and low‐risk groups (Table 3, Supporting Information Table S6). The number of controls in the lowest risk (<1.4% 10‐year risk) and highest risk (≥8%) groups increased from respectively (low: 20 [1.2%], high: 37 [2.2%]) using only the Tyrer–Cuzick model based on classical risk factors to (372 [22.3%], 123 [7.4%]) when mammographic density and the PRS were added; the number of cases increased from (low: 5 [1.2%], high: 17 [4.2%]) to (38 [9.4%], 59 [14.6%]).

**Table 3:** Percentage of cases and controls in 10‐year breast cancer risk groups defined using classical factors (Tyrer–Cuzick (TC) model), mammographic density (D) and SNP143

		Ten‐year risk group (%)				
Risk algorithm	Sample	<1.4%	1.4–3.5%	3.5–5%	5–8%	8%+
TC	Control	20 (1.2%)	1,153 (69.1%)	285 (17.1%)	173 (10.4%)	37 (2.2%)
TC × D	Control	141 (8.5%)	977 (58.6%)	281 (16.8%)	186 (11.2%)	83 (5.0%)
TC × SNP143	Control	293 (17.6%)	862 (51.7%)	247 (14.8%)	182 (10.9%)	84 (5.0%)
TC × SNP143 × D	Control	372 (22.3%)	804 (48.2%)	194 (11.6%)	175 (10.5%)	123 (7.4%)
TC	Case	5 (1.2%)	261 (64.4%)	68 (16.8%)	54 (13.3%)	17 (4.2%)
TC × D	Case	19 (4.7%)	194 (47.9%)	98 (24.2%)	60 (14.8%)	34 (8.4%)
TC × SNP143	Case	25 (6.2%)	171 (42.2%)	84 (20.7%)	79 (19.5%)	46 (11.4%)
TC × SNP143 × D	Case	38 (9.4%)	134 (33.1%)	79 (19.5%)	95 (23.5%)	59 (14.6%)

Sensitivity analysis did not reveal a substantial gain in predictive ability from including SNPs at less than genome‐wide significance for ER‐specific scores (adjusted ΔLR‐χ^2^: ER+ 76.8 (SNPs included with *p* < 10^−5^) *vs*. 70.9 (SNPs included with *p* < 10^−8^); ER−, respectively 13.1 *vs*. 16.6; Table 2, Supporting Information Table S4). There was very little difference in predictive ability for invasive breast cancer (*n* = 323) or DCIS (*n* = 82), where both had fully adjusted aAUC measures approximately 0.64 (see Supporting Information Tables S9 and 10).

## Discussion

Our study showed the ability of a panel of 143 SNPs to further stratify risk of breast cancer when combined with data about classical risk factors and mammographic density. The polygenic score relative risk was well calibrated after adjustment for classical factors and mammographic density and added useful information on risk, as in our earlier study using a panel of 18 SNPs.^16^ Our data also confirm that PRSs are predictive for the risk of both ER‐positive and ER‐negative disease.

Stratifying risk by ER status has several uses in clinical practice. Identifying women at high risk of ER‐positive disease is important because these women can be considered for preventive therapy with selective ER modulators such as tamoxifen when premenopausal, or raloxifene or aromatase inhibitors such as anastrozole and exemestane when postmenopausal.^26^, ^27^ There has also been some consideration of risk‐adapted screening, where women at the lowest risk of poor prognosis breast cancer subtypes are recommended less frequent screening or starting screening at a later age than those at average population risk.^28^ In contrast, higher risk women could receive more frequent screening or the use of more sensitive modalities such as breast magnetic resonance imaging.

The adjusted OR per IQR associated with the panel of 143 SNPs was approximately 2.0. For comparison, we reassessed a SNP score based on 18 loci used previously, and the adjusted OR per IQR was approximately 1.7. We observed diminishing predictive value for the more recently identified SNPs. The most informative 20 SNPs using the overview *p*‐value contributed 61% of the information from all 143 SNPs. In theory, further improvements from additional risk‐associated variants exist but are unlikely to improve predictions substantially and it may be very difficult to achieve much higher relative risks using PRSs than the doubling of risk across the IQR observed here.

This article focused on the ability of PRSs to add to the Tyrer–Cuzick model and mammographic density. The ability of the latter has been assessed in several studies, including PROCAS.^10^, ^11^, ^13^, ^19^, ^29^, ^30^, ^31^ An informative way to compare predictive ability of the domains is to consider the proportion of women who are (accurately) determined to be in high‐risk groups, where more intensive surveillance or prevention measures might be cost‐effective. Table 3 shows that in this sample the number of high‐risk women (controls) is approximately doubled by including mammographic density, and approximately tripled by including SNPs, as earlier predicted.^32^ Thus, each domain can be thought of as providing an equal contribution to risk stratification in general screening population settings.

An important finding of our study is that the PRSs predicted ER− cases as well as ER+. This complements the ability of mammographic density to assess ER‐negative breast cancer,^33^ as well as some of the risk factors in the Tyrer–Cuzick model.^34^ In particular, age, family history of breast cancer^35^ and (premenopause) obesity^36^ have been seen to be associated with ER‐negative breast cancer. On the other hand, certain reproductive and hormonal factors included in the model, such as age at first child, younger age at menarche, hormone replacement therapy as well as proliferative benign disease, have been found to be more associated with hormone‐receptor‐positive disease.^14^, ^37^

Our study has a number of limitations. First, not all the cases were identified after saliva donation, and they might not be fully representative of cases that would arise in prospective cohorts. Second, while the sample was from a population‐based cohort, those included had to consent to join the wider study, and also to subsequently volunteer to provide a saliva sample. There was some evidence that the women who joined the saliva substudy were more likely to have a family history of the disease. However, this is unlikely to lead to meaningful bias because the study included many women at low risk from classical factors, and the main analysis was conditional upon risk assessment based on the Tyrer–Cuzick model and mammographic density. Third, although we considered some different risk scores based on different thresholds on which SNPs to include, we lacked power to assess small changes to risk score, such in our ER‐subtype risk scores that included SNPs in a PRS with *p* < 10^−5^. This has recently been investigated in a much larger study of women with European ancestry.^38^ Predictive performance of their larger panel of 313 SNPs was similar to here but drew on using wholly imputed SNPs that has important barriers for clinical use. Fourth, the measure of mammographic density assessed has only been used in research settings. Fifth, the conclusions relate to Caucasian women attending routine screening and may not necessarily apply to high‐risk groups^21^ or women from different ethnic backgrounds. Finally, the majority of breast cancer cases in our study were identified within 6 years of the entry questionnaire and may not apply to longer‐term risk, where classical risk factors and mammographic density have been validated.^39^ Nonetheless, this is an independent validation of SNPs in a PRS, and none of the cases or controls were used in the discovery analysis used to identify the breast cancer associated SNPs.

In conclusion, PRSs based on the most recent genome‐wide significant SNPs increase predictive ability over the previous SNP score assessed in this cohort. They help to stratify risk by ER status, with implications for risk‐adapted screening and prevention. In combination with classical risk factors and mammographic density, a much greater degree of risk stratification is possible which increases potential benefits from new risk‐adapted screening and prevention strategies.

## Author contributions

Study concept and study design: Evans DGR, Cuzick J, Howell A, Brentnall AR, Newman WG. Data collection and assembly of data: van Veen E, Harkness EF, Byers H, Astley SM, Sampson S, Howell A, Newman WG, Evans DGR. Data analysis and interpretation: Brentnall AR, Rafiq S, van Veen E, Cuzick J and Evans DGR. Manuscript writing and final approval of the manuscript: all authors.

## Supporting information


**Table S1** Risk factors for PROCAS controls in this study (*n* = 1,668), and those not included (*n* = 54,128). The continuous statistics give median (interquartile range).
**Table S2**: Risk factors for PROCAS cases in this study (*n* = 405), and those not included (*n* = 794). The continuous statistics give median (interquartile range).
**Table S3**: Observed and expected number of cases per decile.
**Table S4**: Predictive information in ER‐specific SNP scores and endpoint (all, ER+, ER−) using a more relaxed criteria for choosing SNPs (*p* < 10^−5^). Median (IQR) log SNP score shown for cases and controls. The two values for IQ‐OR (odds ratio per standard deviation), LR‐CHI2 (likelihood ratio chi‐square), calibration and aAUC (concordance index for the SNP score) represent estimates that are adjusted for (*i*) age (study design) and (*ii*) the Tyrer–Cuzick model and mammographic density.
**Table S5**: Predictive information in SNPs, ordered by *p* value from overview data. Median (IQR) shown for cases and controls. The two values for IQ‐OR (odds ratio per interquartile range), LR‐CHI2 (likelihood ratio chi‐square) and aAUC (concordance index for the SNP score) represent estimates that are adjusted for (*i*) age (study design) and (*ii*) the Tyrer–Cuzick model and mammographic density. *SNP18 uses SNPs at the loci from an earlier case–control study that included some women in this study.
**Table S6**: Reclassification matrix for cases and controls
**Table S7**: Number of failures by SNP. CHR: chromosome, position, build 37 chromosome position.
**Table S8**: Summary statistics for each SNP. SNP‐NUM: order of SNP in SNP143 by P‐value from earlier publication; SNP: rs number or name of SNP; CHR: chromosome; POSITION: position in chromosome (build 37 coordinate); RA: risk allele; O/E the observed to expected number of homozygotes assuming HWE; Ph case/ctl corresponding p‐value; RAF onco: expected risk allele frequency; RAF case/ctl: observed allele frequency in cases and controls; OR onco, expected per‐allele odds ratio; OR: observed per‐allele odds ratio; SNP143: whether SNP was used in SNP143 (N, no, blank, yes); SNP18: whether SNP at locus in earlier SNP18 risk score (blank, no, Y, yes).
**Table S9**: Risk factors for cases (by invasive/*in situ* status) and controls in the study. The continuous statistics give median (interquartile range).
**Table S10**: Predictive information in three SNP scores (breast cancer, ER+ breast cancer, ER− breast cancer) and endpoint (all, ER+, ER−), split by invasive/DCIS. Median (IQR) log SNP score shown for cases and controls. The two values for IAR‐OR (odds ratio per standard deviation), LR‐CHI2 (likelihood ratio chi‐square), calibration and aAUC (concordance index for the SNP score) represent estimates that are adjusted for (*i*) age (study design) and (*ii*) the Tyrer–Cuzick model and mammographic density (fully adjusted).
