# Supplementary material for: A case–control evaluation of 143 single nucleotide polymorphisms for breast cancer risk stratification with classical factors and mammographic density
Source: Int J Cancer. 2019 Jul 13;146(8):2122–9. doi: 10.1002/ijc.32541 (PMC7065068; doi:10.1002/ijc.32541)
Supplement: Supplementary file 1 — Table S1 Risk factors for PROCAS controls in this study (n = 1,668), and those not included (n = 54,128). The continuous statistics give median (interquartile range). Table S2: Risk factors for PROCAS cases in this study (n = 405), and those not included (n = 794). The continuous statistics give median (interquartile range). Table S3: Observed and expected number of cases per decile. Table S4: Predictive information in ER‐specific SNP scores and endpoint (all, ER+, ER−) using a more relaxed criteria for choosing SNPs (p < 10−5). Median (IQR) log SNP score shown for cases and controls. The two values for IQ‐OR (odds ratio per standard deviation), LR‐CHI2 (likelihood ratio chi‐square), calibration and aAUC (concordance index for the SNP score) represent estimates that are adjusted for (i) age (study design) and (ii) the Tyrer–Cuzick model and mammographic density. Table S5: Predictive information in SNPs, ordered by p value from overview data. Median (IQR) shown for cases and controls. The two values for IQ‐OR (odds ratio per interquartile range), LR‐CHI2 (likelihood ratio chi‐square) and aAUC (concordance index for the SNP score) represent estimates that are adjusted for (i) age (study design) and (ii) the Tyrer–Cuzick model and mammographic density. *SNP18 uses SNPs at the loci from an earlier case–control study that included some women in this study. Table S6: Reclassification matrix for cases and controls Table S7: Number of failures by SNP. CHR: chromosome, position, build 37 chromosome position. Table S8: Summary statistics for each SNP. SNP‐NUM: order of SNP in SNP143 by P‐value from earlier publication; SNP: rs number or name of SNP; CHR: chromosome; POSITION: position in chromosome (build 37 coordinate); RA: risk allele; O/E the observed to expected number of homozygotes assuming HWE; Ph case/ctl corresponding p‐value; RAF onco: expected risk allele frequency; RAF case/ctl: observed allele frequency in cases and controls; OR onco, expected per‐allele odds rat [file IJC-146-2122-s001.pdf]

Supplementary material to: A case-control evaluation of 143 single nucleotide polymorphisms for breast cancer risk stratification with classic factors and breast density

Adam R. Brentnall, Elke M. van Veen, Elaine F. Harkness, Sajjad Rafiq, Helen Byers, Susan M. Astley, Sarah Sampson, Anthony Howell, William G. Newman, Jack Cuzick, D. Gareth R. Evans

May 29, 2019

Table S1: Risk factors for PROCAS controls in this study (n=1668), and those not included (n=54128). The continuous statistics give median (inter-quartile range).

Risk factor	In this study	Not in study	P	Missing study	Missing Other
(a) Continuous					
Age	60 (54-65)	56 (51-63)	<0.001	0 (0%)	0 (0%)
Age first child (parous)	24 (19-27)	23 (19-27)	0.016	0 (0%)	8 (0%)
BMI	25.9 (23.1-29.8)	26.4 (23.6-30.3)	<0.001	68 (4%)	3728 (7%)
Density (%)	26.0 (14.5-38.7)	24.1 (13.5-37.6)	0.004	0 (0%)	6319 (12%)
Density residual	0.01 (-0.65-0.66)	-0.08 (-0.74-0.64)	0.005	0 (0%)	0 (0%)
TC 10-yr (%)	2.87 (2.26-3.70)	2.65 (2.11-3.44)	<0.001	0 (0%)	0 (0%)
(b) Binary					
First-deg rel	225 (13.5%)	6276 (11.6%)	0.019	0 (0%)	0 (0%)
Parous	1401 (84.0%)	46843 (86.6%)	0.003	1 (0%)	29 (0%)
White	1541 (96.3%)	49233 (94.0%)	<0.001	68 (4%)	1732 (3%)

Table S2: Risk factors for PROCAS cases in this study (n=405), and those not included (n=794). The continuous statistics give median (inter-quartile range).

Risk factor	In this study	Not in study	P	Unknown study	Unknown Not
(a) Continuous					
Age	60 (53-65)	58 (52-64)	0.016	0 (0%)	0 (0%)
Age first child (parous)	24 (19-28)	23 (19-27)	0.5	0 (0%)	0 (0%)
BMI	26.5 (23.9-30.3)	27.1 (24.2-30.9)	0.27	28 (7%)	57 (7%)
Density (%)	29.5 (18.8-42.0)	28.8 (17.5-42.0)	0.5	0 (0%)	88 (11%)
Density residual	0.35 (-0.33-1.02)	0.32 (-0.37-0.96)	0.5	0 (0%)	0 (0%)
TC 10-yr (%)	3.03 (2.35-4.22)	2.86 (2.22-3.93)	0.030	0 (0%)	0 (0%)
(b) Binary					
First-deg rel	65 (16.0%)	121 (15.2%)	0.8	0 (0%)	0 (0%)
Parous	1401 (84.0%)	46843 (86.6%)	0.003	0 (0%)	0 (0%)
White	375 (95.9%)	706 (92.3%)	0.025	14 (3%)	29 (4%)

Table S3: Observed and expected number of cases per decile.

	Cut	N	Expected-Cancer	Observed-Cancer	Expected-OR	Observed-OR
	0.00	208	17	17	0.37	0.37 (0.23-0.57)
10%	0.46	207	23	18	0.53	0.39 (0.25-0.60)
20%	0.59	207	28	24	0.64	0.54 (0.37-0.78)
30%	0.70	207	32	40	0.75	0.99 (0.74-1.29)
40%	0.80	209	36	37	0.85	0.89 (0.66-1.17)
50%	0.90	206	39	37	0.97	0.90 (0.67-1.19)
60%	1.03	207	43	43	1.09	1.08 (0.82-1.39)
70%	1.18	207	49	56	1.27	1.53 (1.21-1.89)
80%	1.38	207	56	61	1.53	1.72 (1.38-2.10)
90%	1.72	208	70	72	2.10	2.18 (1.79-2.60)

Table S4: Predictive information in ER-specific SNP scores and endpoint (all, ER+, ER-) using a more relaxed criteria for choosing SNPs ($P < 10^{-5}$). Median (IQR) log SNP score shown for cases and controls. The two values for IQ-OR (odds ratio per standard deviation), LR-CHI2 (likelihood ratio chi-square), calibration and aAUC (concordance index for the SNP score) represent estimates that are adjusted for (i) age (study design) and (ii) the Tyrer-Cuzick model and mammographic density.

SNP143	ER	Controls	Cases	IQ-OR (95%CI)	LR-CHI2	Calibration (95%CI)	aAUC (95%CI)
SNP-ER-POS	ER+ (353 cases)	-0.18 (-0.53-0.17)	0.12 (-0.23-0.44)	2.07 (1.76-2.44)	84.2	1.04 (0.81-1.28)	0.65 (0.62-0.68)
	– fully adjusted			2.02 (1.72-2.38)	76.8	1.01 (0.77-1.24)	0.65 (0.62-0.68)
SNP-ER-NEG	ER- (39 cases)	-0.09 (-0.30-0.16)	0.16 (-0.13-0.35)	2.10 (1.42-3.11)	13.4	1.61 (0.76-2.47)	0.67 (0.59-0.76)
	– fully adjusted			2.08 (1.40-3.07)	13.1	1.59 (0.74-2.44)	0.67 (0.59-0.76)

Table S5: Predictive information in SNPs, ordered by P value from overview data. Median (IQR) shown for cases and controls. The two values for IQ-OR (odds ratio per inter-quartile range), LR-CHI2 (likelihood ratio chi-square) and aAUC (concordance index for the SNP score) represent estimates that are adjusted for (i) age (study design) and (ii) the Tyrer-Cuzick model and mammographic density. *SNP18 uses SNPs at the loci from an earlier case-control study that included some women in this study.

SNP143	Controls	Cases	IQ-OR (95%CI)	LR-CHI2	Calibration (95%CI)	aAUC (95%CI)
SNP1-20	-0.09 (-0.34-0.16)	0.06 (-0.18-0.32)	1.75 (1.51-2.03)	55.4	1.10 (0.81-1.40)	0.61 (0.58-0.64)
			1.71 (1.47-1.99)	49.8	1.06 (0.76-1.36)	0.61 (0.58-0.64)
SNP21-40	-0.02 (-0.14-0.11)	0.01 (-0.12-0.15)	1.27 (1.10-1.47)	10.6	0.96 (0.38-1.54)	0.55 (0.52-0.58)
			1.25 (1.08-1.45)	8.9	0.89 (0.30-1.48)	0.55 (0.52-0.58)
SNP41-60	-0.02 (-0.12-0.07)	0.00 (-0.11-0.10)	1.21 (1.06-1.38)	7.5	0.99 (0.28-1.70)	0.54 (0.51-0.57)
			1.21 (1.05-1.38)	7.2	0.98 (0.26-1.69)	0.54 (0.51-0.57)
SNP61-80	-0.02 (-0.11-0.08)	0.00 (-0.10-0.09)	1.05 (0.90-1.21)	0.4	0.24 (-0.54-1.02)	0.52 (0.49-0.55)
			1.06 (0.91-1.23)	0.6	0.31 (-0.48-1.11)	0.52 (0.49-0.55)
SNP81-100	0.00 (-0.09-0.07)	0.01 (-0.07-0.09)	1.15 (0.99-1.33)	3.5	0.84 (-0.04-1.72)	0.53 (0.50-0.56)
			1.13 (0.98-1.31)	2.7	0.74 (-0.15-1.63)	0.53 (0.50-0.56)
SNP101-120	-0.02 (-0.10-0.06)	0.01 (-0.07-0.10)	1.39 (1.21-1.60)	22.1	2.20 (1.28-3.12)	0.57 (0.54-0.60)
			1.38 (1.20-1.59)	20.5	2.16 (1.22-3.09)	0.57 (0.54-0.60)
SNP121-143	0.01 (-0.06-0.07)	0.00 (-0.07-0.07)	0.97 (0.84-1.12)	0.2	-0.26 (-1.37-0.86)	0.51 (0.48-0.54)
			0.95 (0.82-1.09)	0.6	-0.44 (-1.55-0.68)	0.52 (0.48-0.55)
SNP1-120	-0.15 (-0.50-0.16)	0.11 (-0.20-0.42)	2.14 (1.83-2.51)	95.2	1.15 (0.91-1.39)	0.65 (0.63-0.68)
			2.09 (1.78-2.45)	86.6	1.11 (0.87-1.36)	0.65 (0.62-0.68)
SNP18*	-0.07 (-0.29-0.15)	0.05 (-0.15-0.27)	1.72 (1.48-2.00)	51.7	1.23 (0.89-1.57)	0.61 (0.58-0.64)
			1.68 (1.44-1.96)	45.9	1.18 (0.83-1.52)	0.61 (0.58-0.64)

Table S6: Reclassification matrix for cases and controls

.	Risk group	<1.4% (combo)	1.4-5% (combo)	5%+ (combo)	Total
Control	<1.4% (TC)	28 (2%)	10 (1%)	1 (0%)	39 (2%)
	1.4-5% (TC)	397 (24%)	859 (51%)	163 (10%)	1419 (85%)
	5%+ (TC)	4 (0%)	72 (4%)	134 (8%)	210 (13%)
	Total	429 (26%)	941 (56%)	298 (18%)	1668 (100%)
Case	<1.4% (TC)	7 (2%)	1 (0%)	0 (0%)	8 (2%)
	1.4-5% (TC)	42 (10%)	188 (46%)	96 (24%)	326 (80%)
	5%+ (TC)	1 (0%)	12 (3%)	58 (14%)	71 (18%)
	Total	50 (12%)	201 (50%)	154 (38%)	405 (100%)
ER- Case	<1.4% (TC)	0 (0%)	0 (0%)	0 (0%)	0 (0%)
	1.4-5% (TC)	6 (15%)	20 (51%)	6 (15%)	32 (82%)
	5%+ (TC)	0 (0%)	0 (0%)	7 (18%)	7 (18%)
	Total	6 (15%)	20 (51%)	13 (33%)	39 (100%)

Table S7: Number of failures by SNP. CHR: chromosome, position, build 37 chromosome position.

SNP name	CHR	POSITION	Case	Control	All
rs616488	1	10566215	0 (0.0%)	1 (0.1%)	1 (0.0%)
rs11249433	1	121280613	0 (0.0%)	1 (0.1%)	1 (0.0%)
rs12405132	1	145644984	0 (0.0%)	1 (0.1%)	1 (0.0%)
chr1_149906413_C_T	1	149906413	0 (0.0%)	1 (0.1%)	1 (0.0%)
rs4971059	1	155148781	0 (0.0%)	0 (0.0%)	0 (0.0%)
rs35383942	1	201437832	1 (0.2%)	3 (0.2%)	4 (0.2%)
rs6678914	1	202187176	0 (0.0%)	0 (0.0%)	0 (0.0%)
rs4951011Âă	1	203766331	10 (2.5%)	8 (0.5%)	18 (0.9%)
rs72755295	1	242034263	0 (0.0%)	0 (0.0%)	0 (0.0%)
rs11552449	1	114448389	0 (0.0%)	0 (0.0%)	0 (0.0%)
rs11117754	1	217206705	0 (0.0%)	0 (0.0%)	0 (0.0%)
rs1707302	1	46600917	0 (0.0%)	1 (0.1%)	1 (0.0%)
rs17426269	1	88156923	0 (0.0%)	0 (0.0%)	0 (0.0%)
rs4245739	1	204518842	0 (0.0%)	0 (0.0%)	0 (0.0%)
rs56212190	1	42168539	0 (0.0%)	0 (0.0%)	0 (0.0%)
rs6660621	1	50838990	0 (0.0%)	0 (0.0%)	0 (0.0%)
rs7544119	1	118215086	0 (0.0%)	0 (0.0%)	0 (0.0%)
rs7554973	1	41387003	0 (0.0%)	0 (0.0%)	0 (0.0%)
chr2_10138552_A_G	2	10138552	4 (1.0%)	7 (0.4%)	11 (0.5%)
chr2_111897506_C_T	2	111897506	1 (0.2%)	35 (2.1%)	36 (1.7%)
chr2_202153920_A_G	2	202153920	0 (0.0%)	0 (0.0%)	0 (0.0%)
rs4442975	2	217920769	0 (0.0%)	0 (0.0%)	0 (0.0%)
chr2_217976869_A_C	2	217976869	0 (0.0%)	0 (0.0%)	0 (0.0%)
rs16857609	2	218296508	0 (0.0%)	1 (0.1%)	1 (0.0%)
rs6725517	2	25129473	76 (18.8%)	220 (13.2%)	296 (14.3%)
rs4577244	2	29120733	0 (0.0%)	0 (0.0%)	0 (0.0%)
rs12710696	2	19320803	0 (0.0%)	0 (0.0%)	0 (0.0%)
rs1550623	2	174212894	0 (0.0%)	0 (0.0%)	0 (0.0%)
rs17726078	2	172974566	0 (0.0%)	0 (0.0%)	0 (0.0%)
rs4849882	2	121243713	0 (0.0%)	2 (0.1%)	2 (0.1%)
rs7587413	2	227207379	0 (0.0%)	0 (0.0%)	0 (0.0%)
chr3_172281430_C_T	3	172281430	0 (0.0%)	0 (0.0%)	0 (0.0%)
rs12493607	3	30682939	0 (0.0%)	0 (0.0%)	0 (0.0%)
rs6796502	3	46866866	1 (0.2%)	0 (0.0%)	1 (0.0%)
rs6762644	3	4742276	0 (0.0%)	0 (0.0%)	0 (0.0%)
chr3_63954649_A_G	3	63954649	0 (0.0%)	0 (0.0%)	0 (0.0%)
rs13066793	3	87037543	3 (0.7%)	12 (0.7%)	15 (0.7%)
rs11706279	3	71517643	0 (0.0%)	0 (0.0%)	0 (0.0%)
rs2871960	3	141121814	0 (0.0%)	2 (0.1%)	2 (0.1%)
rs4973768	3	27416013	0 (0.0%)	0 (0.0%)	0 (0.0%)
rs9833888	3	99723580	0 (0.0%)	0 (0.0%)	0 (0.0%)
rs6828523	4	175846426	0 (0.0%)	0 (0.0%)	0 (0.0%)
rs10022462	4	89243818	0 (0.0%)	0 (0.0%)	0 (0.0%)
rs5743551	4	38807654	0 (0.0%)	0 (0.0%)	0 (0.0%)
rs9790517	4	106084778	0 (0.0%)	0 (0.0%)	0 (0.0%)
rs1432679	5	158244083	0 (0.0%)	0 (0.0%)	0 (0.0%)
rs13162653	5	16187528	0 (0.0%)	0 (0.0%)	0 (0.0%)
rs2012709	5	32567732	0 (0.0%)	0 (0.0%)	0 (0.0%)
rs10472076	5	58184061	0 (0.0%)	0 (0.0%)	0 (0.0%)
rs1353747	5	58337481	2 (0.5%)	2 (0.1%)	4 (0.2%)
rs7707921	5	81538046	0 (0.0%)	0 (0.0%)	0 (0.0%)
kgp3323585	5	56052695	0 (0.0%)	0 (0.0%)	0 (0.0%)
rs10069690	5	1279790	0 (0.0%)	1 (0.1%)	1 (0.0%)
rs10474352	5	90732225	0 (0.0%)	1 (0.1%)	1 (0.0%)

Table S7: Number of failures by SNP (cont.). CHR: chromosome, position, build 37 chromosome position.

SNP name	CHR	POSITION	Case	Control	All
rs10941679	5	44706498	0 (0.0%)	0 (0.0%)	0 (0.0%)
rs13718	5	132441302	0 (0.0%)	0 (0.0%)	0 (0.0%)
rs28416084	5	217640	3 (0.7%)	5 (0.3%)	8 (0.4%)
rs3215401	5	1296255	0 (0.0%)	1 (0.1%)	1 (0.0%)
rs6569648	6	130349119	0 (0.0%)	1 (0.1%)	1 (0.0%)
rs204247	6	13722523	0 (0.0%)	0 (0.0%)	0 (0.0%)
rs3819405	6	16399557	0 (0.0%)	0 (0.0%)	0 (0.0%)
rs11242675	6	1318878	2 (0.5%)	5 (0.3%)	7 (0.3%)
rs17530068	6	82193109	0 (0.0%)	0 (0.0%)	0 (0.0%)
rs2747652	6	152437016	0 (0.0%)	0 (0.0%)	0 (0.0%)
rs3757322	6	151942194	0 (0.0%)	0 (0.0%)	0 (0.0%)
rs4713186	6	28909465	0 (0.0%)	0 (0.0%)	0 (0.0%)
rs7754957	6	81072063	0 (0.0%)	0 (0.0%)	0 (0.0%)
rs9397437	6	151952332	0 (0.0%)	0 (0.0%)	0 (0.0%)
rs9485372	6	149608874	0 (0.0%)	0 (0.0%)	0 (0.0%)
chr7_101546316_C_T	7	101546316	0 (0.0%)	0 (0.0%)	0 (0.0%)
rs4593472	7	130667121	0 (0.0%)	0 (0.0%)	0 (0.0%)
rs720475	7	144074929	0 (0.0%)	0 (0.0%)	0 (0.0%)
chr7_21939032_A_C	7	21939032	0 (0.0%)	1 (0.1%)	1 (0.0%)
rs17156577	7	28356889	0 (0.0%)	1 (0.1%)	1 (0.0%)
rs6964587	7	91630620	0 (0.0%)	0 (0.0%)	0 (0.0%)
rs10232398	7	94227509	0 (0.0%)	0 (0.0%)	0 (0.0%)
rs11977670	7	139942304	0 (0.0%)	2 (0.1%)	2 (0.1%)
rs514192	8	102478959	1 (0.2%)	0 (0.0%)	1 (0.0%)
rs13267382	8	117209548	0 (0.0%)	0 (0.0%)	0 (0.0%)
rs9693444	8	29509616	0 (0.0%)	0 (0.0%)	0 (0.0%)
rs13365225	8	36858483	0 (0.0%)	0 (0.0%)	0 (0.0%)
rs6472903	8	76230301	0 (0.0%)	0 (0.0%)	0 (0.0%)
rs11780156	8	129194641	0 (0.0%)	0 (0.0%)	0 (0.0%)
rs13281615	8	128355618	2 (0.5%)	1 (0.1%)	3 (0.1%)
rs2943559	8	76417937	0 (0.0%)	0 (0.0%)	0 (0.0%)
rs1011970	9	22062134	0 (0.0%)	1 (0.1%)	1 (0.0%)
rs10759243	9	110306115	0 (0.0%)	0 (0.0%)	0 (0.0%)
rs10760444	9	129396434	0 (0.0%)	0 (0.0%)	0 (0.0%)
rs10816625	9	110837073	0 (0.0%)	0 (0.0%)	0 (0.0%)
rs13294895	9	110837176	0 (0.0%)	0 (0.0%)	0 (0.0%)
rs676256Âă	9	110895353	0 (0.0%)	0 (0.0%)	0 (0.0%)
rs7904519	10	114773927	0 (0.0%)	1 (0.1%)	1 (0.0%)
rs11814448	10	22315843	0 (0.0%)	0 (0.0%)	0 (0.0%)
rs704010	10	80841148	0 (0.0%)	0 (0.0%)	0 (0.0%)
rs10995189	10	64278181	0 (0.0%)	0 (0.0%)	0 (0.0%)
rs11199914	10	123093901	0 (0.0%)	0 (0.0%)	0 (0.0%)
rs2380205	10	5886734	0 (0.0%)	0 (0.0%)	0 (0.0%)
rs2912779	10	123337182	0 (0.0%)	0 (0.0%)	0 (0.0%)
rs2981578	10	123340311	0 (0.0%)	0 (0.0%)	0 (0.0%)
rs7072776	10	22032942	0 (0.0%)	0 (0.0%)	0 (0.0%)
rs11820646	11	129461171	0 (0.0%)	0 (0.0%)	0 (0.0%)
rs3817198	11	1909006	0 (0.0%)	0 (0.0%)	0 (0.0%)
rs3903072	11	65583066	0 (0.0%)	0 (0.0%)	0 (0.0%)
rs11246316	11	805712	0 (0.0%)	0 (0.0%)	0 (0.0%)
rs554219	11	69331642	0 (0.0%)	0 (0.0%)	0 (0.0%)
rs75915166	11	69379161	0 (0.0%)	0 (0.0%)	0 (0.0%)
rs206966	12	120832146	0 (0.0%)	1 (0.1%)	1 (0.0%)
rs12422552	12	14413931	0 (0.0%)	0 (0.0%)	0 (0.0%)
rs7297051	12	28174817	0 (0.0%)	1 (0.1%)	1 (0.0%)

Table S7: Number of failures by SNP (cont.). CHR: chromosome, position, build 37 chromosome position.

SNP name	CHR	POSITION	Case	Control	All
rs17356907	12	96027759	0 (0.0%)	0 (0.0%)	0 (0.0%)
rs2464264	12	115835798	4 (1.0%)	0 (0.0%)	4 (0.2%)
rs11571833	13	32972626	0 (0.0%)	0 (0.0%)	0 (0.0%)
rs6562760	13	73957681	0 (0.0%)	0 (0.0%)	0 (0.0%)
rs2236007	14	37132769	24 (5.9%)	62 (3.7%)	86 (4.1%)
rs941764	14	91841069	0 (0.0%)	0 (0.0%)	0 (0.0%)
rs11627032	14	93104072	0 (0.0%)	0 (0.0%)	0 (0.0%)
rs2588809	14	68660428	0 (0.0%)	0 (0.0%)	0 (0.0%)
rs4983386	14	105210207	6 (1.5%)	6 (0.4%)	12 (0.6%)
rs999737	14	69034682	0 (0.0%)	0 (0.0%)	0 (0.0%)
rs2290203	15	91512067	0 (0.0%)	0 (0.0%)	0 (0.0%)
rs4784227	16	52599188	0 (0.0%)	0 (0.0%)	0 (0.0%)
rs17817449	16	53813367	0 (0.0%)	0 (0.0%)	0 (0.0%)
rs11075995	16	53855291	0 (0.0%)	0 (0.0%)	0 (0.0%)
rs28539243	16	54682064	0 (0.0%)	0 (0.0%)	0 (0.0%)
rs11641297	16	87084966	0 (0.0%)	0 (0.0%)	0 (0.0%)
rs13329835	16	80650805	0 (0.0%)	0 (0.0%)	0 (0.0%)
chr17_29221277_C_T	17	29221277	0 (0.0%)	0 (0.0%)	0 (0.0%)
rs72826962	17	40836389	0 (0.0%)	0 (0.0%)	0 (0.0%)
chr17_53205917_A_G	17	53205917	0 (0.0%)	0 (0.0%)	0 (0.0%)
rs745570	17	77781725	0 (0.0%)	0 (0.0%)	0 (0.0%)
rs527616	18	24337424	0 (0.0%)	0 (0.0%)	0 (0.0%)
rs1436904	18	24570667	0 (0.0%)	0 (0.0%)	0 (0.0%)
rs6507583	18	42399590	0 (0.0%)	1 (0.1%)	1 (0.0%)
rs16963205	18	29930576	0 (0.0%)	0 (0.0%)	0 (0.0%)
rs78269692	19	13158277	0 (0.0%)	0 (0.0%)	0 (0.0%)
rs2965183	19	19545696	0 (0.0%)	0 (0.0%)	0 (0.0%)
rs1531212	19	13951830	0 (0.0%)	0 (0.0%)	0 (0.0%)
rs3760982	19	44286513	0 (0.0%)	1 (0.1%)	1 (0.0%)
rs4808801	19	18571141	5 (1.2%)	35 (2.1%)	40 (1.9%)
rs67397200	19	17401404	12 (3.0%)	51 (3.1%)	63 (3.0%)
rs2284378Âă	20	32588095	0 (0.0%)	0 (0.0%)	0 (0.0%)
rs16991615	20	5948227	0 (0.0%)	0 (0.0%)	0 (0.0%)
rs746427	20	48939076	0 (0.0%)	0 (0.0%)	0 (0.0%)
rs2823093	21	16520832	0 (0.0%)	0 (0.0%)	0 (0.0%)
rs6001930	22	40876234	0 (0.0%)	0 (0.0%)	0 (0.0%)
rs73161324	22	42038786	0 (0.0%)	1 (0.1%)	1 (0.0%)
rs132390	22	29621477	0 (0.0%)	1 (0.1%)	1 (0.0%)
rs17879961	22	29121087	0 (0.0%)	0 (0.0%)	0 (0.0%)
rs6001031	22	38556260	0 (0.0%)	0 (0.0%)	0 (0.0%)

Table S8: Summary statistics for each SNP. SNP-NUM: order of SNP in SNP143 by P-value from earlier publication; SNP: rs number or name of SNP; CHR: chromosome; POSITION: position in chromosome (build 37 coordinate); RA: risk allele; O/E the observed to expected number of homozygotes assuming HWE; Ph case/ctl corresponding p-value; RAF onco: expected risk allele frequency; RAF case / ctl: observed allele frequency in cases and controls; OR onco, expected per-allele odds ratio; OR: observed per-allele odds ratio; SNP143: whether SNP was used in SNP143 (N= no, blank=yes); SNP18: whether SNP at locus in earlier SNP18 risk score (blank=no, Y=yes).

SNP-NUM	SNP	CHR	POSITION	RA	O/E ctl	O/E case	Ph ctl	Ph case	RAF onco	RAF ctl	RAF case	OR onco	OR	SNP143	SNP18
1	rs2912779	10	123337182	C	0.99	0.95	0.605	0.331	59	60	53	0.79	0.75		Y
	rs2981578	10	123340311	T	0.99	0.94	0.563	0.255	53	54	52	0.81	0.77	N	
2	rs4784227	16	52599188	T	0.99	1.03	0.626	0.375	24	24	27	1.24	1.20		Y
3	kgp3323585	5	56052695	C	1.01	0.99	0.708	0.748	16	16	18	1.19	1.21		Y
4	rs4442975	2	217920769	T	0.98	0.97	0.442	0.622	50	53	53	0.88	0.78		Y
5	rs75915166	11	69379161	A	1.00	0.99	0.974	0.596	6	5	7	1.30	1.41		Y
6	rs10941679	5	44706498	G	1.03	0.97	0.150	0.542	25	24	24	1.14	1.01		Y
7	rs7297051	12	28174817	T	0.99	0.98	0.717	0.594	24	25	22	0.89	0.86		
8	rs13281615	8	128355618	G	0.97	1.03	0.281	0.541	40	41	44	1.11	1.14		Y
9	rs4973768	3	27416013	T	1.01	0.97	0.693	0.611	47	47	46	1.10	1.32		Y
10	rs9397437	6	151952332	A	1.01	1.00	0.300	0.941	7	6	8	1.20	1.34		Y
11	rs676256Âă	9	110895353	T	0.99	0.97	0.561	0.604	62	64	65	1.10	1.04		
12	rs11249433	1	121280613	G	1.02	1.02	0.496	0.643	40	41	44	1.10	1.10		Y
13	rs10995189	10	64278181	A	1.00	1.00	0.967	0.924	16	15	12	0.88	0.79		Y
14	rs554219	11	69331642	G	1.00	0.99	0.914	0.825	13	12	14	1.21	1.21		
15	rs3757322	6	151942194	G	0.98	0.97	0.465	0.492	32	33	33	1.09	0.97		
16	rs17356907	12	96027759	G	0.99	1.01	0.527	0.706	30	31	28	0.91	0.90		
17	rs2464264	12	115835798	A	1.01	0.95	0.539	0.329	42	42	42	0.92	0.97		
18	rs999737	14	69034682	T	1.01	0.97	0.655	0.536	23	26	24	0.91	0.92		Y
19	chr2_217976869_A_C	2	217976869	C	1.00	1.00	0.595	0.850	5	5	4	0.83	0.75		
20	rs704010	10	80841148	C	1.00	1.02	0.983	0.650	62	58	54	0.92	0.86		Y
21	rs6001930	22	40876234	C	1.00	1.00	0.949	0.831	11	9	11	1.13	1.25		
22	rs1432679	5	158244083	T	1.03	0.92	0.265	0.139	57	56	55	0.93	0.94		
23	chr17_53205917_A_G	17	53205917	G	1.03	0.98	0.191	0.670	30	30	30	0.93	1.01		Y
	rs4808801	19	18571141	G	1.01	1.02	0.500	0.615	35	35	31	0.93	0.83	N	
24	rs13329835	16	80650805	G	1.00	1.02	0.918	0.544	22	21	22	1.08	1.05		
25	rs2747652	6	152437016	C	0.98	1.01	0.467	0.846	53	51	54	1.07	1.11		
26	rs6828523	4	175846426	A	0.99	0.98	0.483	0.373	12	12	11	0.90	0.91		
27	rs16857609	2	218296508	T	0.99	1.04	0.621	0.284	26	26	28	1.08	1.10		
28	rs2943559	8	76417937	G	1.01	1.00	0.528	0.808	8	8	9	1.12	1.09		
29	rs9693444	8	29509616	C	0.99	0.99	0.759	0.876	68	67	66	0.94	0.95		
30	rs17817449	16	53813367	G	1.02	1.02	0.381	0.682	41	37	37	0.94	0.98		
	rs2236007	14	37132769	A	0.99	0.98	0.438	0.548	21	21	20	0.93	0.92	N	

Table S8: Summary statistics for each SNP (cont.)

SNP-NUM	SNP	CHR	POSITION	RA	O/E ctl	O/E case	Ph ctl	Ph case	RAF onco	RAF ctl	RAF case	OR onco	OR	SNP143	SNP18
31	rs6472903	8	76230301	T	1.00	0.98	0.796	0.421	83	82	85	1.08	1.24		
32	rs3215401	5	1296255	AG	1.03	1.03	0.214	0.416	31	31	29	0.94	0.91		
33	rs13365225	8	36858483	G	1.01	0.97	0.464	0.289	18	16	16	0.93	0.99		
34	rs2823093	21	16520832	A	0.98	1.03	0.271	0.470	27	28	26	0.94	0.87		
35	rs616488	1	10566215	G	0.96	0.99	0.097	0.921	33	34	35	0.94	1.06		
36	rs7072776	10	22032942	G	1.01	1.03	0.565	0.494	71	73	73	0.94	1.01		
37	rs4849882	2	121243713	A	1.00	1.00	0.933	0.870	90	90	92	1.10	1.27		
38	rs3817198	11	1909006	C	1.00	1.03	0.930	0.520	31	33	34	1.06	1.05		Y
39	rs10759243	9	110306115	A	1.00	1.00	0.980	0.977	29	28	29	1.06	1.03		
40	rs6762644	3	4742276	G	1.00	0.98	0.901	0.675	39	42	42	1.06	0.99		
41	rs10816625	9	110837073	G	1.00	1.00	0.779	0.744	6	5	5	1.12	1.05		
42	rs11814448	10	22315843	C	1.00	1.00	0.982	0.973	2	1	2	1.20	2.11		
43	rs13294895	9	110837176	T	0.98	0.98	0.140	0.496	18	17	20	1.07	1.15		
44	rs10069690	5	1279790	T	1.02	0.99	0.387	0.798	26	24	24	1.06	1.01		
45	rs11977670	7	139942304	A	1.03	0.96	0.199	0.450	43	43	43	1.05	1.01		
46	rs3760982	19	44286513	G	0.97	1.08	0.264	0.093	54	52	51	0.95	0.90		
47	chr2_202153920_A_G	2	202153920	G	0.99	1.01	0.626	0.842	72	72	72	0.95	1.03		Y
48	rs1011970	9	22062134	T	0.99	0.99	0.537	0.785	17	17	16	1.07	0.91		Y
49	rs11571833	13	32972626	T	1.00	1.00	0.650	0.593	1	1	2	1.31	2.55		
50	rs12422552	12	14413931	C	0.97	0.98	0.161	0.740	26	26	31	1.06	1.29		
51	rs6796502	3	46866866	A	1.00	0.98	0.936	0.450	10	11	10	0.92	0.90		
52	rs527616	18	24337424	G	0.98	0.99	0.447	0.925	62	64	64	1.05	0.99		
53	rs28539243	16	54682064	A	0.98	0.98	0.375	0.655	49	49	48	1.05	0.96		
54	rs1436904	18	24570667	G	0.98	0.91	0.395	0.061	40	40	37	0.95	0.90		
55	chr1_149906413_C_T	1	149906413	C	0.99	1.02	0.839	0.629	40	40	39	1.05	0.95		
56	rs72755295	1	242034263	G	1.00	1.00	0.744	0.823	3	3	4	1.15	1.17		
57	rs11820646	11	129461171	C	1.02	0.99	0.453	0.850	60	58	58	1.05	0.98		
58	rs2588809	14	68660428	C	0.99	1.00	0.662	0.843	84	84	85	0.94	1.04		
59	rs12493607	3	30682939	C	0.99	0.99	0.570	0.774	34	33	33	1.05	0.99		
60	rs2871960	3	141121814	C	1.05	1.03	0.050	0.450	44	44	45	1.05	1.02		
61	rs11780156	8	129194641	T	0.99	1.00	0.590	0.929	17	18	20	1.06	1.13		
62	rs7904519	10	114773927	G	1.02	1.09	0.489	0.060	46	47	49	1.05	1.08		
63	rs6001031	22	38556260	G	1.00	1.01	0.883	0.774	37	39	38	0.95	0.96		
64	rs35383942	1	201437832	T	0.99	1.00	0.436	0.842	6	6	6	1.11	1.01		
65	rs204247	6	13722523	A	0.99	1.02	0.676	0.609	56	58	56	0.96	0.94		
66	rs941764	14	91841069	G	0.98	1.06	0.443	0.166	34	35	33	1.05	0.92		
67	rs7707921	5	81538046	A	1.00	1.01	0.998	0.752	75	73	75	1.05	1.13		

Table S8: Summary statistics for each SNP (cont.)

SNP-NUM	SNP	CHR	POSITION	RA	O/E ctl	O/E case	Ph ctl	Ph case	RAF onco	RAF ctl	RAF case	OR onco	OR	SNP143	SNP18
68	rs6507583	18	42399590	G	1.00	1.01	0.938	0.481	7	9	8	0.92	0.93		
69	rs3903072	11	65583066	T	1.03	1.04	0.207	0.340	47	46	48	0.96	1.06		
	rs6725517	2	25129473	G	1.17	1.14	0.000	0.008	41	45	49	0.95	1.16	N	
70	rs6569648	6	130349119	T	1.01	0.99	0.632	0.881	76	77	76	1.05	0.95		
71	rs17530068	6	82193109	C	1.00	0.97	0.885	0.500	24	23	21	1.05	0.87		
72	rs5743551	4	38807654	C	1.01	0.99	0.680	0.767	25	20	20	1.05	0.96		
73	rs2965183	19	19545696	A	1.00	1.04	0.891	0.359	35	35	33	1.05	0.91		
74	rs11199914	10	123093901	T	1.00	0.97	0.958	0.518	32	32	30	0.96	0.92		
75	rs10232398	7	94227509	A	1.01	1.00	0.469	0.949	73	69	70	0.95	1.05		
76	rs17726078	2	172974566	G	0.96	1.03	0.080	0.567	47	47	48	0.96	1.06		
77	chr7_101546316_C_T	7	101546316	T	0.99	0.99	0.617	0.702	12	13	15	0.93	1.16		
78	rs720475	7	144074929	A	1.00	1.00	0.929	0.894	25	26	23	0.95	0.86		
79	rs11246316	11	805712	G	0.99	1.00	0.787	0.992	51	50	53	1.04	1.13		
80	rs13267382	8	117209548	G	0.99	1.04	0.548	0.368	65	67	65	0.96	0.93		
81	rs4593472	7	130667121	T	1.02	0.97	0.397	0.604	35	36	35	0.96	0.96		
82	rs11627032	14	93104072	C	1.03	0.99	0.135	0.728	26	28	23	0.95	0.78		
83	chr3_63954649_A_G	3	63954649	G	1.01	1.00	0.287	0.843	14	15	15	1.06	1.01		
84	rs10474352	5	90732225	T	1.00	0.98	0.969	0.480	16	16	15	0.94	0.93		
85	rs11552449	1	114448389	T	0.99	1.03	0.579	0.280	17	18	16	1.06	0.86		
86	rs4971059	1	155148781	A	1.01	1.04	0.659	0.357	36	36	36	1.04	1.01		
87	rs9790517	4	106084778	T	0.99	1.04	0.537	0.214	23	21	22	1.05	1.03		
88	rs6964587	7	91630620	T	1.01	0.96	0.780	0.409	39	41	40	1.04	0.97		
89	chr3_172281430_C_T	3	172281430	C	0.98	1.04	0.358	0.244	22	22	23	1.05	1.06		
90	rs745570	17	77781725	G	0.95	0.95	0.035	0.371	50	52	50	0.96	0.93		
91	chr17_29221277_C_T	17	29221277	T	1.01	0.97	0.589	0.428	27	27	27	0.96	1.00		
92	rs9833888	3	99723580	T	1.00	1.01	0.903	0.791	23	22	25	1.05	1.15		
93	rs1550623	2	174212894	A	0.99	1.01	0.587	0.745	84	84	84	1.05	1.01		
94	rs12405132	1	145644984	T	1.04	1.03	0.047	0.420	37	36	36	0.96	0.98		
95	rs7544119	1	118215086	A	1.00	0.95	0.908	0.205	24	24	26	1.05	1.08		
96	rs2290203	15	91512067	A	1.01	1.02	0.534	0.437	20	19	20	0.95	1.02		
97	rs13066793	3	87037543	G	0.99	1.01	0.632	0.519	9	9	8	0.93	0.88		
98	rs16963205	18	29930576	C	1.00	1.00	0.700	0.723	4	3	3	0.91	1.03		
99	rs6562760	13	73957681	G	0.99	1.02	0.760	0.637	77	75	75	1.05	1.02		
100	rs10022462	4	89243818	T	0.94	0.98	0.009	0.796	44	46	47	1.04	1.05		
101	rs78269692	19	13158277	C	1.00	1.00	0.899	0.997	5	5	5	1.10	1.13		
102	rs16991615	20	5948227	A	1.00	1.00	0.994	0.890	6	6	7	1.08	1.07		
103	rs73161324	22	42038786	T	1.00	1.00	0.868	0.779	6	5	7	1.08	1.52		

Table S8: Summary statistics for each SNP (cont.)

SNP-NUM	SNP	CHR	POSITION	RA	O/E ctl	O/E case	Ph ctl	Ph case	RAF onco	RAF ctl	RAF case	OR onco	OR	SNP143	SNP18
104	rs746427	20	48939076	A	1.01	1.03	0.592	0.323	18	19	20	1.05	1.06		
105	rs7754957	6	81072063	A	0.97	1.02	0.188	0.689	54	53	56	1.04	1.15		
106	rs1353747	5	58337481	G	1.00	1.00	0.918	0.904	9	11	8	0.94	0.75		
107	rs17156577	7	28356889	C	1.00	0.99	0.869	0.883	11	11	12	1.06	1.11		
108	rs72826962	17	40836389	T	1.00	1.00	0.974	0.973	1	1	2	1.19	1.96		
109	rs514192	8	102478959	T	1.02	0.98	0.262	0.641	68	68	63	0.96	0.82		
110	rs11117754	1	217206705	G	0.97	0.99	0.156	0.733	21	22	21	0.96	0.91		
111	rs11075995	16	53855291	T	1.02	0.98	0.231	0.609	76	77	76	0.96	0.95		
112	rs10760444	9	129396434	A	1.00	1.07	0.988	0.170	57	55	54	0.96	0.95		
113	rs13718	5	132441302	G	1.01	1.01	0.575	0.687	24	24	23	0.96	0.98		
114	rs10472076	5	58184061	C	1.02	1.00	0.451	0.929	38	38	40	1.04	1.11		
115	rs17879961	22	29121087	G	1.00	1.00	0.883	0.000	1	0	0	1.28	0.00		
116	rs132390	22	29621477	T	1.00	1.00	0.777	0.866	96	96	96	0.91	1.03		
117	rs2012709	5	32567732	T	1.01	0.97	0.555	0.588	47	47	48	1.04	1.03		
118	rs12710696	2	19320803	C	1.04	0.92	0.049	0.095	64	65	61	0.96	0.85		
119	rs1531212	19	13951830	A	1.00	1.06	0.911	0.095	23	24	21	0.96	0.83		
120	rs4713186	6	28909465	C	1.00	1.08	0.931	0.078	40	39	38	1.04	0.95		
	rs67397200	19	17401404	G	1.02	1.03	0.429	0.478	30	28	28	1.04	0.99	N	
121	rs3819405	6	16399557	T	1.03	0.96	0.210	0.450	35	32	33	0.96	1.02		
122	rs17426269	1	88156923	A	0.98	0.99	0.256	0.695	15	17	16	1.05	0.92		
123	rs11641297	16	87084966	T	0.99	0.99	0.613	0.833	25	24	26	0.96	1.15		
124	rs1707302	1	46600917	G	0.97	0.92	0.170	0.090	66	67	68	1.04	1.03		
125	rs206966	12	120832146	T	1.00	1.00	0.859	0.982	16	15	14	1.05	0.92		
126	rs6660621	1	50838990	C	0.98	0.91	0.384	0.071	49	49	49	0.97	0.98		
127	rs7587413	2	227207379	G	1.00	1.02	0.941	0.552	21	20	21	0.96	1.02		
128	rs11706279	3	71517643	C	1.06	1.04	0.021	0.355	48	47	45	0.97	0.95		
129	rs4983386	14	105210207	A	1.00	1.01	0.938	0.792	45	44	46	1.03	1.06		
	chr2_111897506_C_T	2	111897506	C	1.00	0.99	0.727	0.709	6	5	6	1.07	1.17	N	
130	rs13162653	5	16187528	T	1.01	1.00	0.815	0.985	46	44	46	0.97	1.08		
131	chr7_21939032_A_C	7	21939032	A	1.00	0.98	0.887	0.628	32	33	34	0.97	1.03		
132	rs7554973	1	41387003	C	1.03	1.01	0.198	0.748	60	60	59	1.03	0.96		
133	rs9485372	6	149608874	A	0.99	1.02	0.403	0.443	19	18	17	0.96	0.96		
134	rs4951011Âă	1	203766331	G	1.01	1.03	0.561	0.324	16	16	17	1.04	1.05		
135	rs11242675	6	1318878	T	0.97	0.95	0.262	0.310	63	64	61	1.03	0.87		
136	rs4245739	1	204518842	A	1.00	0.96	0.834	0.389	74	72	71	0.97	0.98		
137	rs2380205	10	5886734	T	1.01	1.00	0.651	0.902	44	43	41	0.98	0.92		Y
138	rs56212190	1	42168539	T	1.01	1.00	0.390	0.885	5	4	4	0.95	0.83		

Table S8: Summary statistics for each SNP (cont.)

SNP-NUM	SNP	CHR	POSITION	RA	O/E ctl	O/E case	Ph ctl	Ph case	RAF onco	RAF ctl	RAF case	OR onco	OR	SNP143	SNP18
139	rs28416084	5	217640	A	1.00	1.03	0.871	0.328	12	12	13	1.03	1.15		
140	chr2_10138552_A_G	2	10138552	G	1.01	0.92	0.661	0.133	43	43	43	1.02	1.00		
141	rs2284378Âă	20	32588095	C	1.01	0.97	0.752	0.580	68	65	67	0.99	1.09		
142	rs6678914	1	202187176	A	0.95	1.00	0.041	0.939	41	41	39	0.99	0.90		
143	rs4577244	2	29120733	T	1.01	0.98	0.678	0.706	23	21	23	0.99	1.15		

Table S9: Risk factors for cases (by invasive / in situ status) and controls in the study. The continuous statistics give median (inter-quartile range).

Risk factor	Control (n=1668)	Case (Invasive, n=323)	Case (DCIS, n=82)
(a) Continuous			
Age	60 (54-65)	60 (53-65)	61 (54-64)
Age first child (parous)	24 (19-27)	24 (19-28)	24 (18-27)
BMI	25.9 (23.1-29.8)	26.6 (24.1-30.7)	26.5 (23.5-29.1)
Density (%)	26.0 (14.5-38.7)	30.2 (19.0-41.6)	26.8 (15.9-46.1)
Density residual	0.01 (-0.65-0.66)	0.39 (-0.27-0.93)	0.19 (-0.58-1.19)
TC 10-yr (%)	2.87 (2.26-3.70)	3.08 (2.37-4.10)	2.95 (2.28-4.63)
(b) Binary			
First-deg rel	225 (13.5%)	48 (14.9%)	17 (20.7%)
Parous	1401 (84.0%)	272 (84.2%)	66 (80.5%)
White	1541 (96.3%)	297 (95.8%)	78 (96.3%)

Table S10: Predictive information in three SNP scores (breast cancer, ER+ breast cancer, ER- breast cancer) and endpoint (all, ER+, ER-), split by invasive / DCIS. Median (IQR) log SNP score shown for cases and controls. The two values for IAR-OR (odds ratio per standard deviation), LR-CHI2 (likelihood ratio chi-square), calibration and aAUC (concordance index for the SNP score) represent estimates that are adjusted for (i) age (study design) and (ii) the Tyrer-Cuzick model and mammographic density (fully adjusted).

SNP143	ER	Controls	Cases	IQ-OR (95%CI)	LR-CHI2	Calibration (95%CI)	aAUC (95%CI)
SNP-ALL	All (323 invasive)	-0.14 (-0.50-0.18)	0.09 (-0.21-0.45)	2.14 (1.79-2.55)	76.8	1.11 (0.85-1.37)	0.65 (0.62-0.68)
	– fully adjusted			2.07 (1.74-2.48)	68.9	1.07 (0.81-1.33)	0.64 (0.61-0.68)
	All (82 DCIS)	-0.14 (-0.50-0.18)	0.20 (-0.25-0.41)	2.00 (1.46-2.75)	19.3	1.02 (0.55-1.48)	0.65 (0.58-0.71)
	– fully adjusted			1.93 (1.41-2.65)	17.1	0.96 (0.50-1.43)	0.64 (0.58-0.70)
SNP-ALL	ER+ (288 invasive)	-0.14 (-0.50-0.18)	0.10 (-0.18-0.46)	2.25 (1.86-2.70)	78.7	1.18 (0.91-1.45)	0.66 (0.63-0.70)
	– fully adjusted			2.18 (1.81-2.63)	71.4	1.14 (0.87-1.42)	0.65 (0.62-0.69)
	ER+ (65 DCIS)	-0.14 (-0.50-0.18)	0.20 (-0.25-0.38)	1.84 (1.30-2.60)	11.9	0.89 (0.38-1.40)	0.63 (0.56-0.70)
	– fully adjusted			1.77 (1.24-2.51)	10.3	0.83 (0.32-1.34)	0.62 (0.55-0.69)
SNP-ALL	ER- (26 invasive)	-0.14 (-0.50-0.18)	0.04 (-0.37-0.28)	1.44 (0.84-2.47)	1.8	0.53 (-0.26-1.32)	0.58 (0.47-0.68)
	– fully adjusted			1.42 (0.83-2.44)	1.6	0.51 (-0.28-1.30)	0.57 (0.47-0.68)
	ER- (13 DCIS)	-0.14 (-0.50-0.18)	0.30 (0.20-0.43)	3.12 (1.41-6.88)	8.3	1.66 (0.51-2.82)	0.74 (0.60-0.88)
	– fully adjusted			2.99 (1.35-6.60)	7.7	1.60 (0.44-2.76)	0.74 (0.60-0.87)
SNP-ER+	ER+ (288 invasive)	-0.17 (-0.50-0.17)	0.13 (-0.25-0.41)	2.02 (1.70-2.40)	66.5	1.04 (0.78-1.30)	0.65 (0.61-0.68)
	– fully adjusted			1.97 (1.65-2.35)	61.2	1.01 (0.75-1.27)	0.64 (0.61-0.68)
	ER+ (65 DCIS)	-0.17 (-0.50-0.17)	0.13 (-0.21-0.43)	1.95 (1.39-2.72)	15.5	0.99 (0.49-1.48)	0.64 (0.57-0.72)
	– fully adjusted			1.88 (1.35-2.63)	14.1	0.94 (0.44-1.44)	0.64 (0.57-0.71)
SNP-ER-	ER- (26 invasive)	-0.06 (-0.26-0.15)	0.09 (-0.10-0.33)	1.69 (1.05-2.70)	4.5	1.26 (0.12-2.41)	0.64 (0.53-0.74)
	– fully adjusted			1.70 (1.06-2.72)	4.6	1.28 (0.13-2.42)	0.64 (0.54-0.74)
	ER- (13 DCIS)	-0.06 (-0.26-0.15)	0.33 (0.05-0.43)	3.63 (1.97-6.69)	16.0	3.12 (1.64-4.60)	0.79 (0.68-0.90)
	– fully adjusted			3.64 (1.95-6.76)	16.0	3.12 (1.62-4.63)	0.79 (0.68-0.90)
